# Schizodontic Molarization of Mandibular Premolars: A Case Report of Gemination

**DOI:** 10.4314/ejhs.v34i1.11

**Published:** 2024-01

**Authors:** Harshata Tirtalli Sathish, Sharad Kamat, Mamata Kamat, Rudrayya S Puranik

**Affiliations:** 1 Department of Conservative Dentistry and Endodontics, Bharati Vidyapeeth (Deemed to be university, Pune) Dental College and Hospital, Sangli, Maharashtra; 2 Department of Oral and Maxillofacial Pathology, Bharati Vidyapeeth (Deemed to be university, Pune) Dental College and Hospital, Sangli, Maharashtra

**Keywords:** Case report, Gemination, Bicuspid, Orthopantomography, Dental restoration

## Abstract

**Background:**

Tooth gemination is a single enlarged or joined tooth with a normal tooth count when the anomalous tooth is counted as one. Mandibular second premolars show an elevated variability of crown morphology. Only nine cases of isolated second premolar macrodontia have been reported in the literature.

**Case Description:**

This case report presents the clinical and radiographic findings and conservative treatment of an atypical and rare case of localized bilateral molarization of mandibular second premolars.

**Conclusion:**

Dental professionals should acquire deeper knowledge about anomalies and plan treatment carefully to avoid unexpected complications during dental procedures caused by morphological ignorance.

## Introduction

Anomalies in odontogenesis give rise to changes in the quantity, size, or structure of teeth. Developmental irregularities such as Gemination, Twinning, or Schizodontism impact tooth shape and are sometimes mistakenly associated with macrodontia or fusion (1). Gemination, specifically, is identified as a tooth shape anomaly wherein a single tooth germ attempts to divide through invagination, resulting in a large tooth with a bifid crown, typically possessing a single root and root canal. Despite this anomaly, the tooth count remains normal when affected tooth is considered as one unit. (2)

Gemination may lead to the formation of a premolar with a molar-like appearance, particularly in the mandibular second premolar region, albeit this occurrence is infrequent (1). This phenomenon is also referred to as “molarization of premolars” (3). Macrodontia refers to a comparatively uncommon morphoanatomical anomaly that characterizes dental gigantism, where the tooth's body exhibits a notable increase in size while the roots remain comparatively smaller (4).

As a result of apical prolongation, the affected teeth exhibit a proportional combination of shortened roots and enlarged pulp chambers. Although the exact cause of this condition remains unknown, it is believed that both genetic and acquired factors may play a role in its development (4).

The occurrence of macrodontia in premolars induced by a unique form of gemination has been infrequently documented. To date, literature reports include only nine cases of isolated macrodontia affecting second premolars, with only five of them exhibiting a bilateral presentation. In this context, we present a rare case involving bilateral premolar molarization in a 32-year-old male patient.

## Case Report

A 32-year-old male patient sought attention at the Department of Conservative Dentistry and Endodontics, complaining of pain in the lower right and left posterior areas of the jaw persisting for one week. The patient had no pertinent family or medical history. External examination revealed no abnormalities. Upon intraoral inspection, dental caries was evident on the occlusal surfaces of the mandibular right and left second premolar and second molar.

The patient exhibited localized recession, along with the presence of plaque and calculus around the mandibular left second premolar. Angle's class I molar relation was observed. Both the mandibular right and left second premolars displayed an unusual ovoid molariform crown featuring an irregular crescent-shaped fissure with a convex aspect towards the lingual side. Clinically, both premolars presented, on average, with a mesiodistal diameter of 10 mm and a buccolingual diameter of 9 mm when fully erupted and occluded, showcasing multiple cusps.

**Figure 3:**
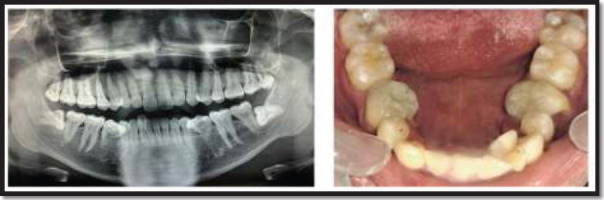
Pre-operative OPG of the patient and Post-operative clinical image of the mandibular jaw, after scaling and restoration

To confirm the diagnosis of the anomaly, an intraoral periapical radiograph was taken, revealing an abnormal size and shape of the tooth with a single short tapering root. Additionally, an OPG was conducted to ascertain the presence of such an anomaly in other quadrants. Remarkably, the described anomaly was isolated to the lower quadrants of the jaw, specifically affecting the mandibular right, and left second premolars. The OPG also revealed an over-retained deciduous incisor and an impacted upper canine (Fig. 3). A conclusive diagnosis of gemination and dentinal caries was established for the mandibular right and left second premolars. Subsequent oral prophylaxis was performed, and composite restorations were applied to address the carious involvement. The patient received education regarding the anomaly and its potential future implications.

## Discussion

The mammalian class exhibits a diverse array of formations falling under the category of “tooth morphology.” Disturbances in odontogenesis give rise to alterations in the quantity, dimensions, or structure of teeth. When the size and structure of teeth exhibit features that deviate from the generally accepted range of normality, they are labelled as anomalies. Gemination, for instance, occurs when a single tooth bud's attempt to divide proves unsuccessful. Neville et al. defined gemination as a singular enlarged tooth or a fused (double) tooth, where the tooth count remains normal when the anomalous tooth is considered as one, as observed in our case. Dugmore introduced the term “macrodont molariform premolars” to describe enlarged premolars that bear a resemblance to molars in terms of their size. The term “molariform” is used because these premolars, being multitubercular, share similarities with molars (1).

The exact cause of gemination remains uncertain, but potential factors include trauma, vitamin deficiencies, systemic disorders, and specific genetic predispositions. Grover and Lorton propose that the origin may involve local metabolic disruptions occurring during the morphodifferentiation of the tooth germ. Additionally, there appears to be a hereditary tendency for this condition. It can manifest in both non-syndromic individuals and those with various syndromes, including insulin-resistant diabetes, pineal hyperplasia with hyperinsulinism, facial hemi hyperplasia, KBG syndrome, Otodental syndrome, Ekman-Westborg and Julin trait syndrome, 47 XYY syndrome, Schinzel-Giedion syndrome, Dubowitz syndrome, and Cockayne's syndrome.

The premolars' molar-like structure involves a reduction in the single vestibular cusp, with small additional cusps resembling shoulders. This results in an appearance akin to that of a mandibular first molar. Anomalies in tooth form, shape, and size stem from disruptions during the morpho differentiation stage of development, although the precise cause of dental anomalies remains unknown. The identification of specific patterns of associated dental issues may be linked to distinct genetic and environmental factors contributing to various sub phenotypes of dental anomalies (2).

Hill (1955) initially documented a molariform premolar at this site. In 1967, Primack documented the initial case of bilateral macrodontia involving mandibular second premolars, with both teeth being unerupted. Subsequently, a year later, Hermel et al. reported a case of bilateral macrodontia in an erupted mandibular second premolar.

Gemination is categorized into two types: Partial cleavage, known as true gemination, and Complete cleavage, referred to as twinning. Based on clinical and radiographic observations, it is reasonable to classify the present case as twinning. The disproportionate conical shape and diminutive root size in comparison to the oversized crown create an image resembling a mushroom. The tooth exhibits a small tapering root supporting an immense crown. Both the mandibular right and left second premolars display a mesiodistal diameter of 10 mm and a buccolingual diameter of 9 mm, surpassing the normal dimensions of 7 and 8 mm, respectively.

Clinically, the existence of a geminated tooth and the presence of a groove can lead to caries and an increased accumulation of plaque. In our patient, the anomaly led to issues such as plaque accumulation, recession, and caries affecting the implicated teeth. However, for cases involving permanent dentition, the preferred treatment can be tailored to meet the patient's specific needs. Some reported approaches include endodontic treatment followed by surgical crown division. Alternatively, extraction with prosthetic replacement is advised in certain instances, along with mesio-distal reduction of tooth structure (2). Our patient, not being concerned about aesthetic problems, underwent essential treatments like oral prophylaxis and restoration.

Further exploration in the form of additional investigations, such as Cone Beam Computed Tomography (CBCT), could be considered. CBCT, with its ability to provide three-dimensional images of dental anomalies and their surrounding areas in all three planes, can be instrumental in formulating an effective treatment plan. Comprehensive knowledge of macrodontia is valuable for identifying potential systemic illnesses or syndromes linked to this condition (5).

In conclusion, renowned philosopher, psychologist, and physician William James asserted that delving into the study of abnormalities is the most effective means of comprehending what is considered normal. Dental professionals should strive to gain a profound understanding of anomalies and meticulously plan treatments to prevent unforeseen complications arising from a lack of awareness of morphological variations during dental procedures.

## Figures and Tables

**Figure 1:**
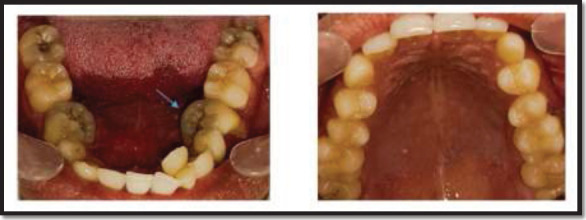
Intra-oral Pre-operative photographs of mandibular and maxillary jaw, the blue arrow showing the Molariform appearance with the mandibular right and left second premolar)

**Figure 2:**
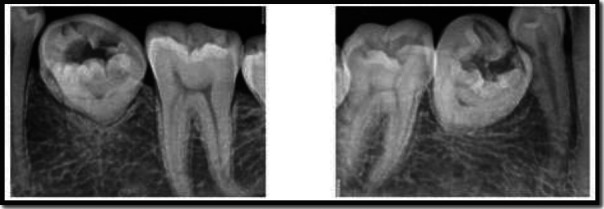
Intraoral periapical radiographs of the patient depicting unusual morphology of mandibular right and left second premolar
